# Severe Factor XII Deficiency in a Patient with Spontaneous Coronary Artery Dissection

**DOI:** 10.3390/ijms27073154

**Published:** 2026-03-31

**Authors:** Artemis Zormpa, Efrosyni G. Nomikou, Dimitrios Kalantzis, Georgios Apergis, Virginia Zouganeli, Efthymia G. Pavlou, Despina Mavridou, Georgios Katsadiotis, Loukianos S. Rallidis, Styliani Kokoris

**Affiliations:** 1 Laboratory of Hematology and Blood Bank Unit, “Attikon” University General Hospital, Medical School, National and Kapodistrian University of Athens, 124 62 Athens, Greece; artemis.zormpa@gmail.com (A.Z.); kaldim70@yahoo.gr (D.K.); mdepy@ymail.com (D.M.); gkatsadiotis@gmail.com (G.K.); 2 Blood Bank and Hemophilia Unit, Hippokration Hospital, 115 27 Athens, Greece; efrosyni.nomikou@gmail.com (E.G.N.); pavlou@hippocratio.gr (E.G.P.); 3 Department of Molecular Diagnosis, “Hippokration” General Hospital, 115 27 Athens, Greece; geast6@hotmail.com; 4 2nd Department of Cardiology, “Attikon” University General Hospital, Medical School, National and Kapodistrian University of Athens, 124 62 Athens, Greece; virginia_noa@yahoo.gr (V.Z.); lrallidis@gmail.com (L.S.R.)

**Keywords:** factor XII, factor 12, FXII, F12, SCAD, spontaneous artery dissection, cardiac arrest, thrombosis, vascular injury, bleeding, coagulation, homozygous variant, gene, point mutation, fibrinolysis, aPTT, haematology, hematology

## Abstract

Factor XII (FXII) deficiency is a coagulation factor disorder inherited in an autosomal recessive manner that causes prolonged activated partial thromboplastin time (aPTT). Although it is clinically benign without additional bleeding risk, it has been associated with paradoxical thrombosis. We report the case of a 38-year-old woman who presented with cardiac arrest secondary to spontaneous coronary artery dissection (SCAD) in the absence of identifiable risk factors. Laboratory investigations revealed an isolated prolonged aPTT, which was corrected with mixing studies, and a severe FXII deficiency with activity levels < 1%. Molecular analysis identified two homozygous *FXII* gene variants: the 46C>T polymorphism and the c.619G>C (p.Ala207Pro) variant. The two variants are considered clinically benign, although the combination of both in a single individual has not been previously reported. The coexistence of FXII deficiency in this case may be of clinical and hypothesis-generating interest.

## 1. Introduction

Factor XII (FXII), also known as Hageman factor, is one of the thirteen traditional clotting factors involved in thrombus formation during blood vessel injury to prevent bleeding. It plays an important role in the intrinsic pathway of the coagulation cascade (contact activation system) that ultimately leads to fibrin clot formation. Secondary functions include activation of the kinin-kallikrein system and fibrinolysis by converting plasminogen to plasmin [1].

Factor XII deficiency is a rare autosomal recessive disorder that causes prolonged activated partial thromboplastin time (aPTT) in vitro with no additional risk of bleeding [2]. The vast majority of individuals with Factor XII deficiency remain asymptomatic throughout their lives, due to other compensatory mechanisms of coagulation, such as the activation of the extrinsic pathway of coagulation (tissue factor activation) [3].

In rare cases, FXII deficiency can lead to paradoxical thrombosis. The suggested mechanisms involve the overactivation of the extrinsic coagulation pathway and impaired fibrinolysis, although the exact pathophysiology is not adequately understood [4].

In this case report, we describe a patient with an undiagnosed severe FXII deficiency in whom two homozygous FXII variants were identified, who presented with cardiac arrest secondary to spontaneous coronary artery dissection.

## 2. Detailed Case Description

A 38-year-old woman with a past medical history of severe iron deficiency anemia, gestational diabetes and 15-pack-year smoking history, presented in the emergency department with sudden onset, severe chest pain radiating to her left arm. The patient reported only one previous pregnancy three years earlier, complicated by gestational diabetes. No prior hemostasis screening or known coagulation disorders were reported. During private transport to the hospital, the patient lost consciousness and developed agonal breathing. Upon arrival at the Emergency Department, she was already in cardiac arrest with a shockable rhythm of ventricular fibrillation.

The patient was intubated and received a total of 8 cycles of cardiopulmonary resuscitation as per the European Council Resuscitation Guidelines, before achieving return of spontaneous circulation (ROSC). An electrocardiogram (ECG) demonstrated sinus rhythm with significant ST-segment elevation in the inferior leads and reciprocal ST-segment depression in the anterolateral leads. A focused cardiac ultrasound revealed hypokinesia of the inferior and posterior walls of the left ventricle.

The patient was transferred to the intensive care unit (ICU). Repeat ECG showed no persistent ischemic changes but a new incomplete right bundle branch block (RBBB). Serial high-sensitivity cardiac troponin-T measurements were consistent with acute myocardial infarction, increasing from 26 pg/mL to 3016 pg/mL within 2 h.

**Figure 1:**
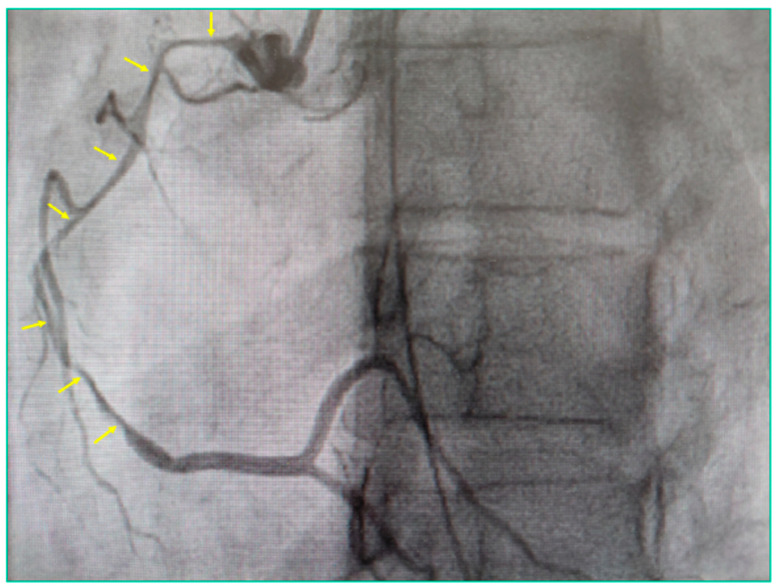
Coronary angiogram with contrast injection into the right coronary artery (RCA) shows. a long, severe diffuse narrowing extending from the ostium to the mid and distal segments of the RCA (yellow arrows), with no visible intimal flap. This appearance is consistent with type 2 spontaneous coronary artery dissection of RCA.

An urgent coronary angiogram was performed and revealed severe narrowing of the right coronary artery (RCA), specifically a long, narrow, tubular lumen without the presence of an obvious flap consistent with type 2 spontaneous coronary artery dissection (SCAD). Given the typical angiographic pattern observed, no additional imaging (e.g., optical coherence tomography) was deemed necessary and was considered potentially dangerous for the patient. Findings regarding the remaining coronary arteries were unremarkable (Figure 1).

The patient was managed conservatively with vasopressors and acetylsalicylic acid. Repeat transthoracic echocardiography demonstrated a normal-sized left ventricle with severe diffuse hypokinesia and a severely impaired left ventricular ejection fraction (LVEF) of 25%.

The patient underwent an extensive laboratory workup to investigate possible causes for SCAD, when an isolated and severely prolonged activated partial thromboplastin time (aPTT) was first observed. INR and prothrombin time were normal. The aPTT values remained elevated throughout her hospital admission, ranging between 149.70 and 219.00 s (normal range: 24–39 s). The patient was not receiving any anticoagulant medication before or during admission that could have interfered with these measurements.

Further evaluation for known risk factors associated with SCAD was unremarkable. Autoimmune, endocrine and connective tissue disease screening were negative. Comprehensive whole-body computed tomography imaging revealed no evidence of fibromuscular dysplasia or other vascular abnormalities. Due to the markedly prolonged aPTT, the patient was referred to the Thrombosis and Hemostasis Department for further diagnostic evaluation.

Mixing studies (mixing the patient’s plasma with normal pooled plasma) are the first diagnostic tool in investigating an isolated prolonged aPTT. The combination of two mixing studies—measured at time 0 h and at 120 min following incubation at 37 °C—allows for the differentiation between the presence of a lupus anticoagulant, a coagulation factor inhibitor, or a coagulation factor deficiency. In our patient, mixing studies corrected the prolonged aPTT to 25 s at 0 h and 27.6 s after 120 min, excluding the presence of a lupus anticoagulant or a coagulation factor inhibitor.

The coagulation factor deficiencies that are unequivocally associated with an isolated prolongation of aPTT are deficiencies of factor VIII, factor IX, factor XI, factor XII and factor XIII. Subsequent testing at our institution confirmed severely impaired FXII with activity levels of <1% (normal range 60–150%). Von Willebrand factor (vWF) activity levels were within normal range, while vWF antigen and FVIII:C levels were slightly increased. Thrombophilia screening (Protein C, Free protein S, Antithrombin AT, FV Leiden, FII G20210A, Lupus Anticoagulant) was negative, apart from heterozygosity for the MTHFR C677T mutation, which is considered clinically insignificant. Homocysteine levels were within normal limits. Anticardiolipin antibodies and antibodies to b2-glycoprotein (IgG and IgM) were also negative.

The patient subsequently underwent molecular testing of the entire *FXII* gene (14 exons, 13 introns, and flanking regions) via Sanger sequencing at the Department of Molecular Diagnosis at an external institution, which revealed two distinct variants:

1. Nt46 (C>T) homozygous variant (T/T genotype)
  The FXII variant 46 (C>T) (FXII 46C>T promoter polymorphism-rs1801020) is a common genetic variant in the promoter region of the FXII gene at nucleotide (nt) 46. The 46C/T polymorphism generates an alternative initiation codon (ATG) for mRNA translation, leading to a frameshift and subsequent truncation of the protein, which results in impaired recognition of the translation site. Homozygous T/T carriers typically have lower circulating levels of Factor XII compared to those with the common C/C or C/T genotype, although all genotypes are considered benign without causing any disease symptoms [5,6] (see Supplementary Material).
2. Nt619 of exon 7 (G>C) homozygous variant
  The analysis identifies a G-to-C substitution at the relevant nucleotide position (e.g., c.619G>C). The (FXII): c.619G>C (p.Ala 207Pro) rs17876030 variant is associated with FXII deficiency. This variant has been primarily described in large case studies from big diagnostic centers, where the presence of FXII deficiency coincides with a normal clinical phenotype. These findings, along with its high allele frequency, lead to its classification as benign [7,8,9,10,11,12] (see Supplementary Material).

The presence of two homozygous variants in a single individual represents a unique finding that could be associated with the markedly prolonged activated partial thromboplastin time (aPTT) observed in the context of severe factor XII deficiency. However, a causal relationship cannot be established based on the available data, and additional functional or confirmatory studies were not performed at the time to further investigate this potential association.

In view of the molecular findings, the patient’s parents also underwent assessment of factor XII activity levels. Both parents demonstrated reduced FXII activity, more specifically 43% in the father and 24% in the mother (reference range, 60–150%). Reduced FXII activity in the parents is compatible with carrier status. Potential heterozygosity was not further investigated, as both parents declined molecular testing. No information regarding shared ancestry was available.

The patient showed gradual clinical improvement and was eventually discharged after 30 days of inpatient hospital stay following implantation of a dual-chamber cardioverter-defibrillator. Follow-up echocardiography demonstrated an increase in LEVF from 25% to 45%. In addition, she was initiated on acetylsalicylic acid 100 mg once daily and metoprolol 25 mg twice daily. The patient has remained clinically stable since discharge.

## 3. Discussion

In this report, we present a case of severe FXII deficiency in an otherwise healthy, asymptomatic adult who presented with cardiac arrest secondary to SCAD, with the rare coexistence pattern of two homozygous variants in the *FXII* gene.

SCAD is a multifactorial condition of poorly understood pathophysiology, presenting as acute coronary syndrome, especially in young to middle-aged women without traditional atherosclerotic risk factors. It is strongly linked to fibromuscular dysplasia, accounting for over 70% of cases, or other connective tissue disorders affecting the integrity of coronary vessel walls. Hormonal or stress triggers, such as pregnancy or infection, are common predisposing factors [13].

SCAD results from compression of the true coronary artery wall, causing cardiac muscle ischemia from reduced blood flow and oxygen delivery. The proposed mechanism is the formation of a hematoma secondary to spontaneous bleeding that causes compression and narrowing of the vessel [13]. The hematoma can either form from a rupture of the vessel wall and bleeding into a false lumen between the intima and media layers of the vessel wall (commonly a flap can be seen during coronary angiography) or a spontaneous bleeding of the vasa vasorum, causing the formation of the hematoma directly within the media (long, tubular, narrow lumen during coronary angiography) [14]. This second mechanism was likely the cause of SCAD in our patient.

Histopathological evidence from post-mortem studies in patients with SCAD revealed that the false lumen becomes thrombosed and the myocardial tissue shows marked coagulative necrosis as well as interstitial edema [15].

Factor XII (FXII, Hageman factor) is a serine protease produced by the liver, traditionally known for its role in initiating the intrinsic pathway of coagulation [16]. Mutations of FXII are generally inherited in an autosomal recessive manner. In vivo, complete FXII deficiency is associated with a markedly prolonged aPTT without increased risk of bleeding, while its biological role is being re-evaluated in the context of thrombosis and inflammation [1]. Clinical reports and experimental models suggest that such individuals may be paradoxically at an increased risk of thrombotic events [17].

**Figure 2:**
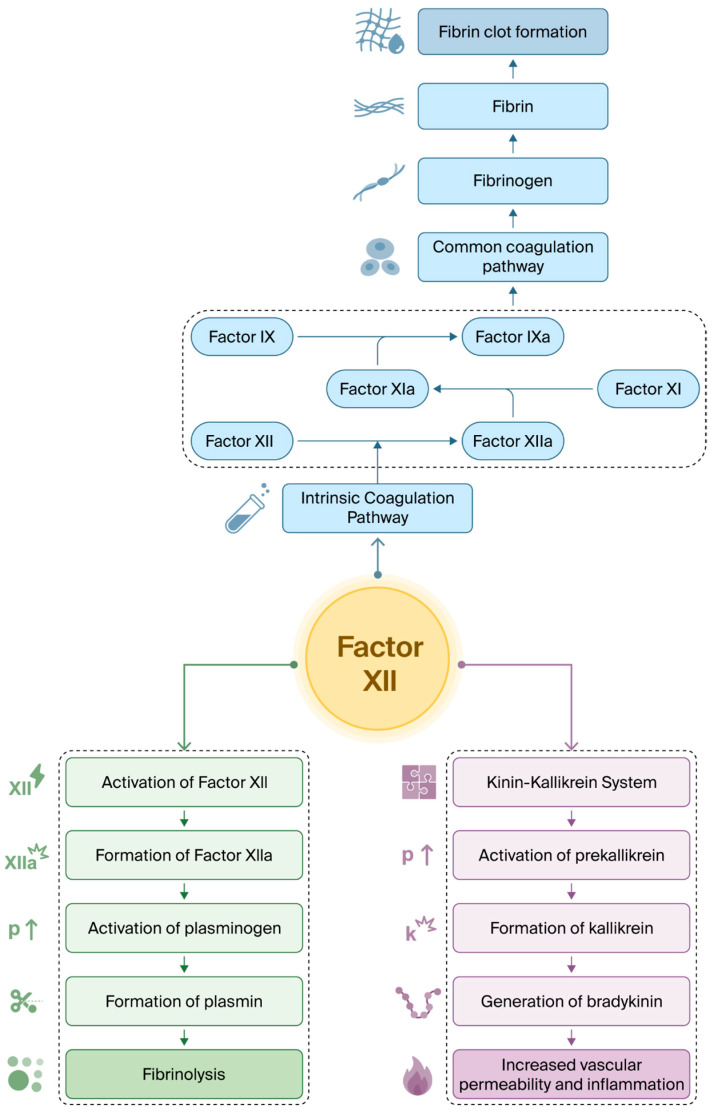
Contact activation of FXII leads to its conversion to activated FXII (FXIIa), initiating the intrinsic coagulation pathway via conversion of factor XI (FXI) to factor XIa (FXIa), which in turn converts factor IX (FIX) to factor IXa (FIXa). FIXa activates downstream pathways in the common coagulation pathway and ultimately leads to fibrin clot formation (blue). FXII facilitates the conversion of plasma prekallikrein (PK) to kallikrein (PKa), leading to the release of bradykinin, which promotes inflammation and increases vascular permeability (purple). FXII also links to fibrinolysis by facilitating plasminogen activation to plasmin, resulting in fibrin degradation (green). Together, FXII functions as a molecular hub integrating coagulation, inflammatory signalling, and fibrinolysis.

FXII has multiple biological roles. Primarily, it initiates the intrinsic pathway (contact pathway) of the coagulation cascade by binding to negatively charged surfaces or by proteolytic activation of endothelial cells via the kinin-kallikrein system [18]. As part of the intrinsic pathway, FXII converts to its activated form, FXIIa, which in turn activates factor XI and eventually results in the formation of thrombin (Figure 2) [19]. However, due to compensatory mechanisms via overactivation of the extrinsic pathway of coagulation, the bleeding risk is ameliorated. In vivo studies of FXII-knockout mice have demonstrated no increased bleeding risk, as blood coagulation relies primarily on tissue factor and the extrinsic coagulation pathway [20].

The role of the extrinsic coagulation pathway in thrombus formation, and its overactivation in FXII deficiency, is one of the proposed mechanisms leading to thrombosis in such patients [21]. The activated form of FXII, FXIIa, also plays a significant role in fibrinolysis by converting plasminogen to plasmin, which is a central enzyme involved in clot breakdown [22]. Therefore, impaired FXII activity reduces fibrinolysis.

Despite the aforementioned proposed mechanisms, the exact pathway of FXII induced thrombosis has not been fully understood. Findings suggesting an increased risk of thrombosis in individuals with FXII deficiency have mainly been reported in cases involving homozygous mutations associated with markedly reduced FXII levels [23]. Interestingly, Haj A. et al., in the largest study on FXII deficiency, demonstrated that heterozygous variants of FXII with moderately low circulating FXII levels were protective against thrombotic events [24].

Multiple variants in the *FXII* gene have been described in the literature. However, homozygous variants in individuals with FXII deficiency are uncommon, as most affected individuals are heterozygous for a single variant. To our knowledge, the presence of two distinct homozygous FXII variants in a single individual, as observed in our patient, is extremely rare and has not been previously reported. Both variants are common in the general population and are considered clinically benign. While their combined effect could have contributed to the markedly reduced FXII levels (<1%), the available data do not support a direct causal relationship, and other mechanisms are likely involved. In the absence of parental genotyping and further functional analyses, it remains a consideration for future investigation.

## 4. Conclusions

This case illustrates the coexistence of two homozygous *FXII* gene variants and severe FXII deficiency in a single individual who presented with cardiac arrest secondary to SCAD. While the literature does not establish a direct causal link between FXII deficiency and SCAD, isolated reports have described associations between FXII defects and arterial or venous thrombotic events. However, these observations are not consistent throughout the reports and may reflect confounding risk factors. Nonetheless, the role of FXII in coagulation, fibrinolysis, and inflammation highlights biological pathways that could affect vascular wall integrity and repair, inflammation, as well as thrombus formation, all of which are mechanisms involved in the development of SCAD. The current understanding of SCAD emphasizes specific risk factors, while coagulation abnormalities represent an area of interest and possibly further study.

## Data Availability

The original contributions presented in this study are included in the article. Further inquiries can be directed to the corresponding author.

## Supplementary Materials

The following supporting information can be downloaded at: https://www.mdpi.com/article/10.3390/ijms27073154/s1.
