# Supplementary material for: Severe Factor XII Deficiency in a Patient with Spontaneous Coronary Artery Dissection
Source: Int J Mol Sci. 2026 Mar 31;27(7):3154. doi: 10.3390/ijms27073154 (PMC13073069; doi:10.3390/ijms27073154)
Supplement: Supplementary file 1 [file ijms-27-03154-s001.zip › ijms-4189452-supplementary.pdf]

Supplementary material

1. HGVS nomenclature data F12, 46C-T (rs1801020)

Location: Cytogenetic: 5q35.3 5: 177409531 (GRCh38)

<u>Nucleotide</u>

NM_000505.4:c.-4T>C
NC_000005.10:g.177409531A>G
NC_000005.9:g.176836532A>G
NG_007568.1:g.5046T>C
LRG_145:g.5046T>C
LRG_145t1:c.-4T>C

<u>Allele frequency</u>

The Genome Aggregation Database (gnomAD), exomes 0.65197

Trans-Omics for Precision Medicine (TOPMed) 0.65725

The Genome Aggregation Database (gnomAD) 0.67118

1000 Genomes Project 30x 0.53076

Exome Aggregation Consortium (ExAC) 0.65458

The Genome Aggregation Database (gnomAD) 0.66266

NHLBI Exome Sequencing Project (ESP) Exome Variant Server 0.69337

1000 Genomes Project 0.52756

The Genome Aggregation Database (gnomAD), exomes 0.71377

<u>Classification :</u>

Benign, Likely benign (7 out of 11 submissions contributed to this classification)

References

[6] Houlihan LM, Davies G, Tenesa A, Harris SE, Luciano M, Gow AJ, et al. Common variants of large effect in F12, KNG1, and HRG are associated with activated partial thromboplastin time. *Am J Hum Genet.* 2010;86(4):626-631. PMID: 20303064.

2. HGVS nomenclature data for Ala207Pro variants

Nucleotide

NM_000505.4:c.619G>C

NC_000005.10:g.177404825C>G

NC_000005.9:g.176831826C>G

NG_007568.1:g.9752G>C

LRG_145:g.9752G>C

LRG_145t1:c.619G>C

Protein

NP_000496.2:p.Ala207Pro

LRG_145p1:p.Ala207Pro,

P00748:p.Ala207Pro

Allele frequency

Trans-Omics for Precision Medicine (TOPMed) 0.95443

The Genome Aggregation Database (gnomAD) 0.96486

The Genome Aggregation Database (gnomAD) 0.96534

1000 Genomes Project 0.93950

1000 Genomes Project 30x 0.93973

NHLBI Exome Sequencing Project (ESP) Exome Variant Server 0.97690

dbSNP: rs17876030

Classification :

Benign (9 out of 11 submissions contributed to this classification)

References

[7] National Center for Biotechnology Information (NCBI). ClinVar: RCV000360167 [Internet]. Bethesda (MD): National Library of Medicine (US); [cited 2026 Mar 10]. Available from: https://www.ncbi.nlm.nih.gov/clinvar/RCV000360167/ (accessed on 10 March 2026)

[8] Clinical Genome Resource (ClinGen). CA3581437 [Internet]. Bethesda (MD): National Institutes of Health; [cited 2026 Mar 10]. Available from: https://search.clinicalgenome.org/kb/allele/CA3581437 (accessed on 10 March 2026)

[9] National Center for Biotechnology Information (NCBI). ClinVar: RCV000252303 [Internet]. Bethesda (MD): National Library of Medicine (US); [cited 2026 Mar 10]. Available from: https://www.ncbi.nlm.nih.gov/clinvar/RCV000252303/ (accessed on 10 March 2026)

[10] National Center for Biotechnology Information (NCBI). ClinVar: RCV000264675 [Internet]. Bethesda (MD): National Library of Medicine (US); [cited 2026 Mar 10]. Available from: https://www.ncbi.nlm.nih.gov/clinvar/RCV000264675/ (accessed on 10 March 2026)

[11] National Center for Biotechnology Information (NCBI). ClinVar: RCV001711545 [Internet]. Bethesda (MD): National Library of Medicine (US); [cited 2026 Mar 10]. Available from: https://www.ncbi.nlm.nih.gov/clinvar/RCV001711545/ (accessed on 10 March 2026)

[12] UniProt Consortium. UniProt variant VAR_014336 [Internet]. Cambridge (UK): UniProt; [cited 2026 Mar 10]. Available from: https://www.uniprot.org/variants/VAR_014336 (accessed on 10 March 2026)
